# The Need for Objective Physical Activity Measurements in Routine Bariatric Care

**DOI:** 10.1007/s11695-022-06165-y

**Published:** 2022-06-23

**Authors:** Ellen Kuipers, Josien Timmerman, Marc van Det, Miriam Vollenbroek-Hutten

**Affiliations:** 1 Department of Surgery, Hospital Group Twente Almelo/Hengelo, 7609 PP Almelo, The Netherlands; 2 Biomedical Signals and Systems, Faculty of Electrical Engineering, Mathematics and Computer Science, University of Twente, 7522 NB Enschede, The Netherlands; 3 ZGT Academy, Hospital Group Twente Almelo/Hengelo, 7609 PP Almelo, The Netherlands

**Keywords:** Physical activity, Sedentary behavior, Bariatric surgery, Accelerometer, Self-reports, Physical behavior profiling, Personalized recommendations

## Abstract

**Purpose:**

This study aims to (1) quantify physical behavior through self-reports and sensor-based measures, (2) examine the correlation between self-reported and sensor-based physical activity (PA) and (3) assess whether bariatric patients adhere to PA guidelines.

**Methods:**

A Fitbit accelerometer was used to collect minute-to-minute step count and heart rate data for 14 consecutive days. Total physical activity levels (PAL), moderate-to-vigorous intensity physical activity (MVPA) and sedentary behavior (SB) were used to quantify physical behavior. Self-reported PA was assessed with the International Physical Activity Questionnaire (IPAQ). To analyze the association between sensor-based and self-reported PA, Spearman’s correlation was used. A minimum of 150 MVPA minutes per week was considered as compliance with the PA guidelines.

**Results:**

Fitbit data of 37 pre- and 18 post-surgery patients was analyzed. Participants averaged 7403 ± 3243 steps/day and spent most of their time sedentary (832 min, IQR: 749 – 879), especially in prolonged periods of ≥ 30 min (525, IQR: 419 – 641). Median MVPA time was 5.6 min/day (IQR: 1.7 – 10.6). Correlations between self-reported and sensor-based MVPA and SB were respectively 0.072 and 0.455. Only 17.1% was objectively adherent to MVPA guidelines ≥ 150 min/week, while 94.3% met the guidelines in case of self-reports.

**Conclusion:**

PA quantification confirmed that bariatric patients are highly sedentary and rarely engage in MVPA, despite a relatively high daily step count. Moreover, bariatric patients are not able to assess MVPA and moderately their SB by self-reports. Our results indicate the need for sensor-based PA monitoring in routine bariatric care.

**Graphical abstract:**

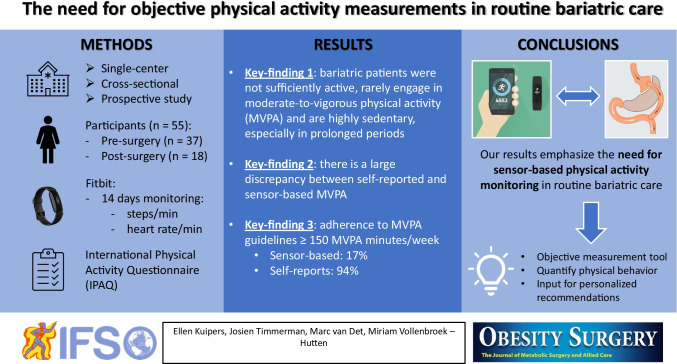

**Supplementary Information:**

The online version contains supplementary material available at 10.1007/s11695-022-06165-y.

## Introduction

The benefits of physical activity (PA) after bariatric surgery on weight loss outcomes have been extensively studied [1]. Studies show that sufficient PA leads to an improvement in cardiorespiratory endurance capacity and muscle function, a better quality of life, a positive effect on mental health and less premature mortality [2–4]. At least 150 min per week of moderate intensity PA for post-bariatric patients is recommended by the Obesity Management Task Force of the European Association for the Study of Obesity to achieve and maintain successful weight loss and reduce the risk of weight regain [5]. Independent of sufficient PA, high levels of sedentary behavior (SB) are recognized as an independent risk factor for developing chronic diseases [6], with more time spent sedentary associated with negative cardio-metabolic health effects in the general population [7].

So far, most studies in the bariatric population used self-reported measures (questionnaires) to capture PA [1]. However, questionnaires are prone to recall bias [8], and persons with obesity are even more likely to overreport their PA levels compared to individuals without obesity, shown by a large discrepancy between self-reported and sensor-based measures [9, 10]. Together, this suggests that questionnaires do not provide reliable information regarding PA levels in the bariatric population.

Sensor-based PA studies in the bariatric population are increasingly being performed [11–15]. However, studies that applied sensors to investigate PA in bariatric patients focused mainly on one component of PA, for example a one-time assessment of post-surgery moderate-to-vigorous intensity physical activity (MVPA) [11], on pre- to postoperative changes in steps [16, 17], sedentary time [16] or MVPA [17–19], or to which extent MVPA influences weight loss outcomes at 6 [15, 20], 12 [20] and 18 [15] months post-surgery. To our best knowledge, no studies were conducted that investigate the holistic physical activity profile of bariatric patients considering the different domains simultaneously (total physical activity levels (PAL), MVPA and SB) and in conjunction with self-reported measures. This is considered important to come to better understanding of their physical activity behavior and through this to more personalized recommendations in clinical practice. This is additionally supported by the fact that, despite PA recommendations, most studies show no increase in pre- to post-surgery PA levels expressed in steps and MVPA using sensor-based assessments [13, 16, 19], whereas those using self-reported questionnaires do [18, 21].

Therefore, this study aims to (1) quantify physical behavior through self-reports and sensor-based measures in terms of PAL, MVPA and sedentary time, (2) investigate whether self-reported MVPA and SB by the IPAQ correlate to accelerometer-based MVPA and SB and (3) assess to which extent bariatric patients adhere to the Obesity Management Task Force of the European Association for the Study of Obesity PA guidelines for both self-reported and sensor-based data. In addition, differences in abovementioned aspects between the pre- and-post-surgery group were explored.

## Methods

### Study Design

A cross-sectional prospective observational cohort study was performed at the obesity center of Hospital Group Twente (ZGT) Almelo/Hengelo, a high-volume bariatric center in The Netherlands. The study protocol was reviewed and approved by the local medical ethics committee (local registration number 2020–07), and all patients signed informed consent.

### Setting and Participants

Patients scheduled for bariatric surgery or patients who underwent bariatric surgery 6 months before were recruited at the outpatient clinic of the obesity center of ZGT between January and June 2021. Inclusion criteria were: (1) sufficient understanding of the Dutch spoken and written language and (2) internet access at home. Exclusion criteria were: (1) inability to walk which was not directly related to obesity and (2) participation in a diet- or intervention program not part of the bariatric care pathway of the obesity center in ZGT.

### Procedures

Eligible participants were approached following a preoperative consult at the outpatient clinic or a postoperative group meeting by the first author (E.K.), a junior researcher (A.J.) or a student (T.V.), who provided verbal and written information about the study. After informed consent was obtained, instructions were given about the procedure and the use of the Fitbit. Participants were asked to wear the accelerometer on the non-dominant wrist for 14 consecutive days except during battery charging and swimming/showering in case of a non-waterproof Fitbit. Two weeks of monitoring was chosen, because previous research has shown that at least 7 days of monitoring is required to reliably assess physical inactivity [22]. Participants were asked to perform their normal, daily routine. They were not blinded for the number of steps taken. To ensure the availability of the minute-to-minute data, participants were instructed to download the Fitbit application on their smartphone and synchronize the Fitbit every 6 days via Bluetooth to transfer the data to the Fitbit dashboard. MVPA data was not visible for the participants on the Fitbit dashboard. After 14 days of monitoring, participants completed the International Physical Activity Questionnaire (IPAQ). The accelerometer and questionnaires were given to the participants after instruction and returned by post at the end of the study period.

### Data Collection and Handling

Demographics and—for post-surgery participants—operative data were collected from medical records. Weight and height were measured at the outpatient clinic at the time of inclusion. Collected patient characteristics included obesity-related comorbidities and self-reported PA measured using the IPAQ.

#### Physical Behavior—Fitbit

Participants were asked to wear a Fitbit Charge HR, Charge 2 or Inspire 2 accelerometer (Fitbit Inc., San Francisco, CA, USA). The Fitbit accelerometer is a wireless, wearable activity tracker that measures accelerations in the x, y and z-axis. Fitbit data was expressed in steps per minute and heart rate per minute. Data collected by the Fitbit is stored on a server in the USA. In line with the General Data Protection Regulation, an email address was created by the first researcher (E.K.) without identifiable personal information of the participant. As a result, only the number of steps taken and the heart rate per minute were stored on the server in the USA. Raw Fitbit data were processed by an algorithm written in MATLAB version R2021a (The Mathworks, Inc. Natick, MA, USA). A valid day consists of ≥ 10 h (600 min) of data during waking hours (i.e. 6.00 AM to 12.00 PM) [23]. A minimum of seven valid days was required for analysis, including at least two weekend days [22].

#### PA Measures

**Fig. 1:**
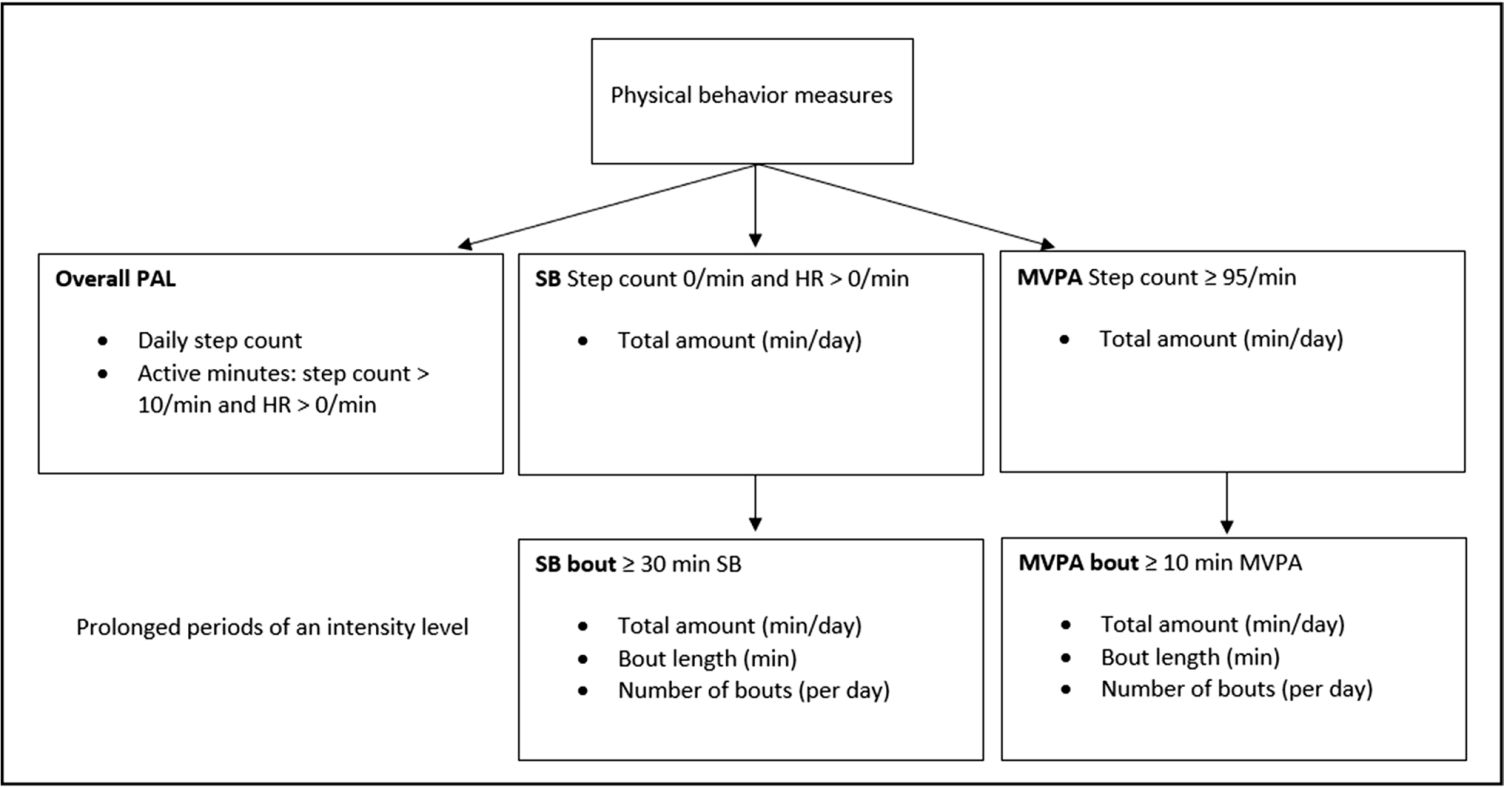
Sensor-based physical behavior measures

Several measures were derived from the accelerometer. The dimensions of interest were PAL, SB and MVPA (Fig. 1). For the definitions and a detailed description, see Appendix.

Non-wearing time was identified by a step count of 0 per minute in combination with a heart rate of 0 per minute.

#### Physical Behavior—IPAQ

Self-reported PA was assessed with the IPAQ. The IPAQ is a 27-item self-reported measure of PA which reflects activities in the previous 7 days according to different domains: occupational, transport, domestic activities and leisure time. Frequency (measured in days per week) and duration (measured in minutes per day) are collected for specific types of activity, namely sitting, walking, moderate intensity activities and vigorous intensity activities. Total activity was defined as the total time per week spent walking, moderately active and vigorously active. The IPAQ has demonstrated acceptable reliability and validity [24]. Data cleaning and processing were performed according to the IPAQ scoring protocol [25].

### Statistical Analysis

Statistical analysis was performed using SPSS version 24 (SPSS Inc., Chicago, IL, USA). Normality of continuous data was tested with the Kolmogorov–Smirnov test or Shapiro–Wilk test. Baseline characteristics and PA measures were expressed as means with standard deviations (SD) for parametric continuous data or frequencies and percentages for categorical data. Median and interquartile range (IQL) were reported for nonparametric continuous data. Spearman’s correlation was performed to determine the association between self-reported PA and sensor-based PA for: (1) minutes of MVPA per day and (2) minutes of SB per day. Under- or overestimation of time spent in MVPA was defined as the discrepancy between sensor-based and self-reported MVPA. The percentage of patients who complied with the PA guidelines (≥ 150 MVPA min/week) was calculated for both self-reported and sensor-based measurements.

Differences in baseline characteristics and physical behavior measures between preoperative and 6 months postoperative patients were analyzed using the independent Student’s *t* test, the Mann–Whitney *U* test and Fisher’s exact or Chi-square test for parametric continuous data, nonparametric continuous data and categorical data, respectively. For all statistical analyses, a *p* value < 0.05 was taken as the threshold of statistical significance.

## Results

### Participants

From the 70 participants who consented to participate, three participants refrained from participation during the study period. Twelve participants were excluded, due to insufficient Fitbit data, resulting in 55 participants with valid accelerometer data. Nine IPAQ questionnaires were not available, and 11 questionnaires were excluded. In 35 participants, both IPAQ and accelerometer data were available.

Demographics of the included participants are presented in Table 1. The median age of our study population was 50 years (IQR: 39 – 54) with a baseline BMI of 41.5 kg/m^2^ (IQR: 39.2 – 44.4). Most participants were female (73%). The One Anastomosis Gastric Bypass (OAGB) was the most performed bariatric procedure (87%). Dyslipidemia (75%) and osteoarthrosis (53%) were the most prevalent obesity-related comorbidities. There were no significant differences in baseline characteristics between the pre-surgery and 6 months post-surgery group.

**Table 1:** Participant characteristics

	Total (***n*** = 55)	Pre-surgery (***n*** = 37)	Post-surgery (***n*** = 18)	*p*
Age, years, median [IQR]	50.0 [39.0 – 54.0]	49.0 [38.5 – 53.0]	51.0 [43.5 – 55.3]	0.25
Gender, ***N*** (%)				0.53
Female	40 (72.7)	28 (75.7)	12 (66.7)	
Male	15 (27.3)	9 (24.3)	6 (33.3)	
Baseline BMI, kg/m^2^, median [IQR]	41.5 [39.2 – 44.4]	41.8 [39.8 – 44.3]	40.6 [37.8 – 45.8]	0.63
Post-surgery BMI, kg/m^2^, median [IQR]	N.A	N.A	31.6 [29.9 – 36.5]	N.A
%EWL, median [IQR]*	N.A	N.A	52.3 [43.9 – 67.2]	N.A
%TWL, median [IQR]*	N.A	N.A	22.5 [19.2 – 25.4]	N.A
Baseline comorbidities, ***N*** (%)				
T2DM	15 (27.3)	9 (24.3)	6 (33.3)	0.53
Hypertension	23 (41.8)	14 (37.8)	9 (50.0)	0.56
OSAS	20 (36.4)	12 (32.4)	8 (44.4)	0.55
Osteoarthrosis	29 (52.7)	20 (54.1)	9 (50.0)	1.00
GERD	26 (47.3)	19 (51.4)	7 (38.9)	0.41
Dyslipidemia	41 (74.5)	28 (75.7)	13 (72.2)	1.00
Type of surgery, ***N*** (%)				0.10
SG	0 (0.0)	0 (0.0)	0 (0.0)	
RYGB	4 (7.3)	1 (2.7)	3 (16.7)	
OAGB	48 (87.3)	34 (91.9)	14 (77.8)	
Re-sleeve gastrectomy	1 (1.8)	1 (2.7)	0 (0.0)	
Redo RYGB	1 (1.8)	0 (0.0)	1 (5.6)	
Redo OAGB	1 (1.8)	1 (2.7)	0 (0.0)	

*IQR* interquartile range, *BMI* body mass index, *EWL* excess weight loss, *TWL* total weight loss, *T2DM* type 2 diabetes mellitus, *OSAS* obstructive sleep apnea syndrome, *GERD* gastro-esophageal reflux disease, *SG* sleeve gastrectomy, *RYGB* Roux-en-Y gastric bypass, *OAGB* one anastomosis gastric bypass

*Percentage excess weight loss (%EWL) was calculated using the formula: [(preoperative weight – current weight) / (preoperative weight – ideal weight)] × 100. Ideal weight implies achieving a target weight listed as BMI of 25 kg/m^2^; and percentage total weight loss (%TWL) by the formula: (weight loss / initial weight) × 100

### Accelerometer-Based Physical Behavior

In 55 participants, there were 928 monitoring days. During analysis, 74 days with less than 600 min of data were removed, resulting in 854 valid days of data. Median number of valid monitoring days was 15 (IQR: 14 – 16).

#### Total Group

Accelerometer-based physical behavior is illustrated in Table 2. Mean step count per day was 7403 ± 3243. Participants were active during 154 (IQR: 125 – 230) min per day. The majority of time was spent sedentary (832 min/day, IQR: 749 – 879), of which most of the time in bouts (525 min/day, IQR: 419 – 641). Mean number of sedentary bouts per day was 8.0 ± 1.7, with a mean bout length of 67.1 ± 7.7 min. Median MVPA time was 5.6 min per day (IQR: 1.7 – 10.6) with almost no MVPA bout per day (0.1, IQR: 0 – 0.3). On average, participants accomplished a valid 10-min MVPA bout once in every 2.2 days.

**Table 2:** Accelerometer-based physical behavior

	Total (***n*** = 55)	Pre-surgery (***n*** = 37)	Post-surgery (***n*** = 18)	*p*
PAL				
Step count, mean (SD)	7403 (3243)	6736 (2947)	8778 (3470)	0.027
Minimum steps/day, median [IQR]	3030 [2123 – 4515]	2872 [1987 – 4113]	4435 [2491 – 6077]	0.021
Maximum steps/day, median [IQR]	11,861 [8049 – 15,723]	9674 [6955 – 14,098]	15,348 [11,415 – 19,420]	0.003
Active minutes per day, median [IQR]	154 [125 – 230]	151 [116 – 205]	193 [145 – 237]	0.173
SB				
SB, min/day, median [IQR]	832 [749 – 879]	835 [770 – 893]	798 [734 – 863]	0.132
SB bouts per day, mean (SD)	8.0 (1.7)	8.3 (1.7)	7.5 (1.6)	0.114
SB bout length in minutes, mean (SD)	67.1 (7.7)	66.8 (7.0)	67.8 (9.1)	0.680
Sedentary bout minutes per day, median [IQR]	525 [419 – 641]	537 [431 – 653]	485 [393 –606]	0.197
MVPA				
MVPA, min/day, median [IQR]	5.6 [1.7 – 10.6]	3.8 [1.6 – 7.2]	10.5 [6.0 – 35.4]	0.001
MVPA bouts per day, median [IQR]	0.1 [0 – 0.3]	0 [0 – 0.1]	0.3 [0.0 – 0.9]	< 0.001
MVPA bout length in minutes, median [IQR]*	16.4 [14.6 – 22.5]	17.3 [13.2 – 19.8]	16.1 [14.9 – 23.3]	0.663
MVPA bout minutes per day, median [IQR]	0.8 [0.0 – 5.3]	0.0 [0.0 – 2.5]	3.4 [1.5 – 16.3]	0.001

*PAL* overall levels of physical activity*, SD* standard deviation, *IQR* interquartile range, *SB* sedentary behavior, *MVPA* moderate-to-vigorous physical activity

*Calculation in patients with a MVPA bout (total; *n* = 29, pre-surgery; *n* = 15 and post-surgery; *n* = 14)

#### Pre- vs. Post-Surgery Group

Mean step count per day was significantly lower in the pre-surgery group (6736 ± 2947), compared to the post-surgery group (8778 ± 3470, *p* = 0.027). Moreover, both minimum and maximum number of steps per day were significantly higher in the post-surgery group (minimum steps: post-surgery group 4435, IQR: 2491 – 6077 vs. pre-surgery group 2872, IQR: 1987 – 4113, *p* = 0.021; maximum steps: post-surgery group 15,348, IQR: 11,415 – 19,420 vs. pre-surgery group 9674, IQR: 6955 – 14,098, *p* = 0.003). Both the pre- and post-surgery group were highly sedentary (pre-surgery group 835 min/day, IQR: 770 – 893 and post-surgery group 798 min/day, IQR: 734 – 863) with no significant differences between both groups in total sedentary time, number of sedentary bouts, sedentary bout length and sedentary bout minutes per day. Median MVPA minutes per day was 3.8 (IQR: 1.6 – 7.2) in the pre-surgery group, which is approximately one-third of the time spent in MVPA per day in the post-surgery group (10.5 min, IQR: 6.0 – 35.4, *p* = 0.001). Overall, the post-surgery group spent significantly more minutes in MVPA and MVPA bouts.

**Fig. 2:**
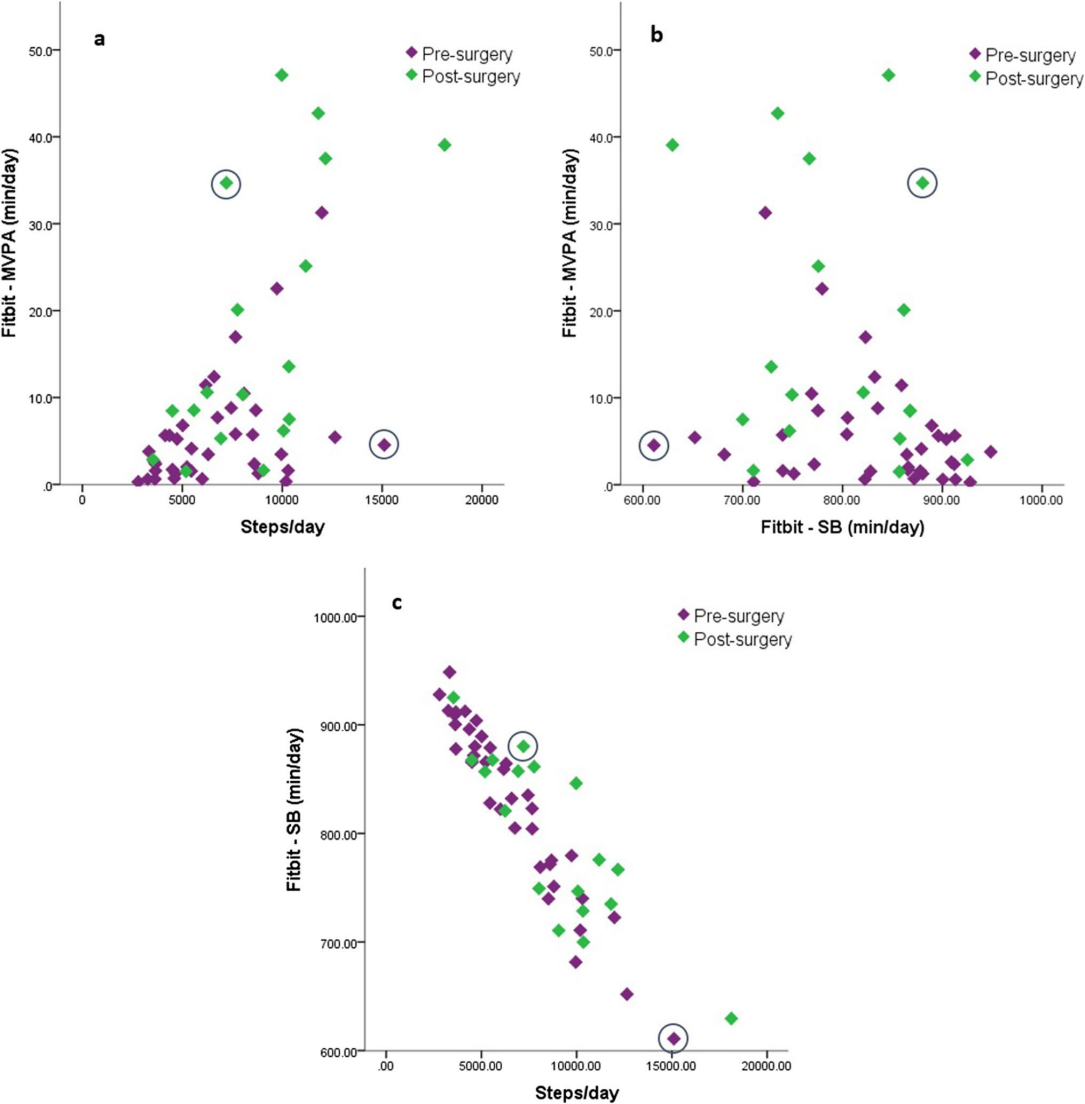
Individual variation of accelerometer-based physical behavior. (**a**) Steps per day vs. MVPA in minutes per day; (**b**) sedentary time in minutes per day vs. MVPA in minutes per day; (**c**) steps per day vs. sedentary time in minutes per day

The overall data presented above show large standard deviations within each PA dimension, meaning large between-subject variability. In addition to this single dimension variability, also the variability of the combination of the three dimensions revealed a great variability and remarkable activity behavioral patterns in and between individuals as is shown in Fig. 2.

For example, there are individuals with a high daily step count (≥ 10.000 per day), limited sedentary time (compared to the total study population), but almost no time spent in MVPA compared to the average of the group. On the other hand, there are also individuals with a low daily step count (≤ 10.000 per day), high levels of SB and high levels of time spent in MVPA (Fig. 2). Also, participants with low numbers of sedentary bouts are not necessarily the individuals with the longest sedentary bouts.

### Self-Reported PA

#### Total Group

In the 35 included IPAQ questionnaires, self-reported MVPA time was 161 min per day (IQR: 53 – 243), and sedentary time was 402 min per day (IQR: 300 – 600) (Table 3).

**Table 3:** Self-reported PA

	Total (***n*** = 35)	Pre-surgery (***n*** = 25)	Post-surgery (***n*** = 10)	*p*
MVPA, min/day, median [IQR]	161 [53 – 243]	141 [51 – 233]	184 [100 – 272]	0.584
SB, min/day, median [IQR]	402 [300 – 600]	446 [313 – 660]	313 [293 – 426]	0.093

*MVPA* moderate-to-vigorous physical activity, *IQR* interquartile range, *SB* sedentary behavior

#### Pre- vs. Post-Surgery Group

There was no significant difference in self-reported MVPA in minutes per day between the pre- and post-surgery group (pre-surgery 141, IQR: 51 – 233 vs. post-surgery 184, IQR: 100 – 272). Reported sedentary time was shorter in the post-surgery group (median 313, IQR: 293 – 426 vs. pre-surgery group 446, IQR: 313 – 660), but this was not statistically significant (*p* = 0.093).

### Correlation Self-Reported and Accelerometer-Based PA

**Fig. 3:**
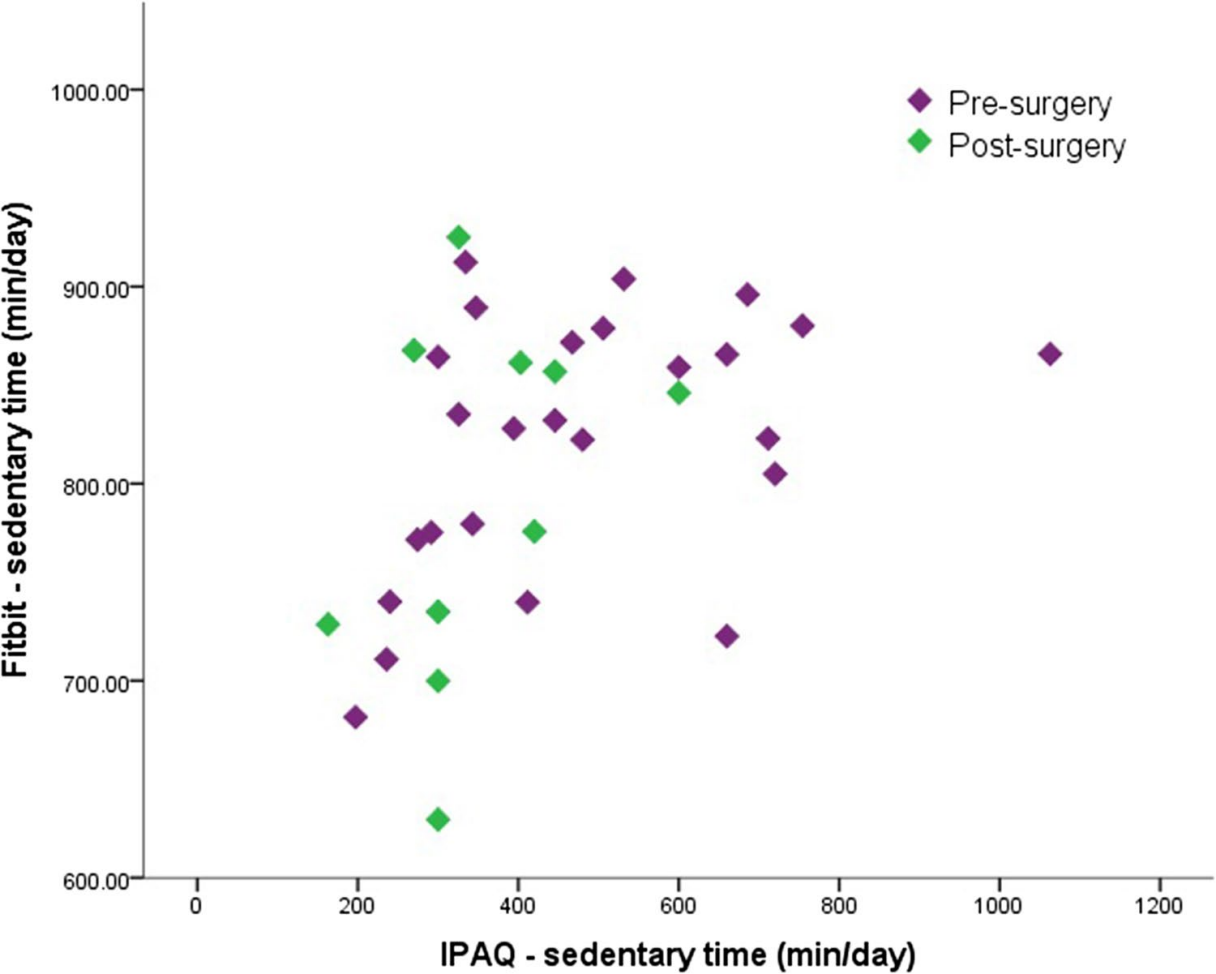
Self-reported and accelerometer-based sedentary time in minutes per day

**Fig. 4:**
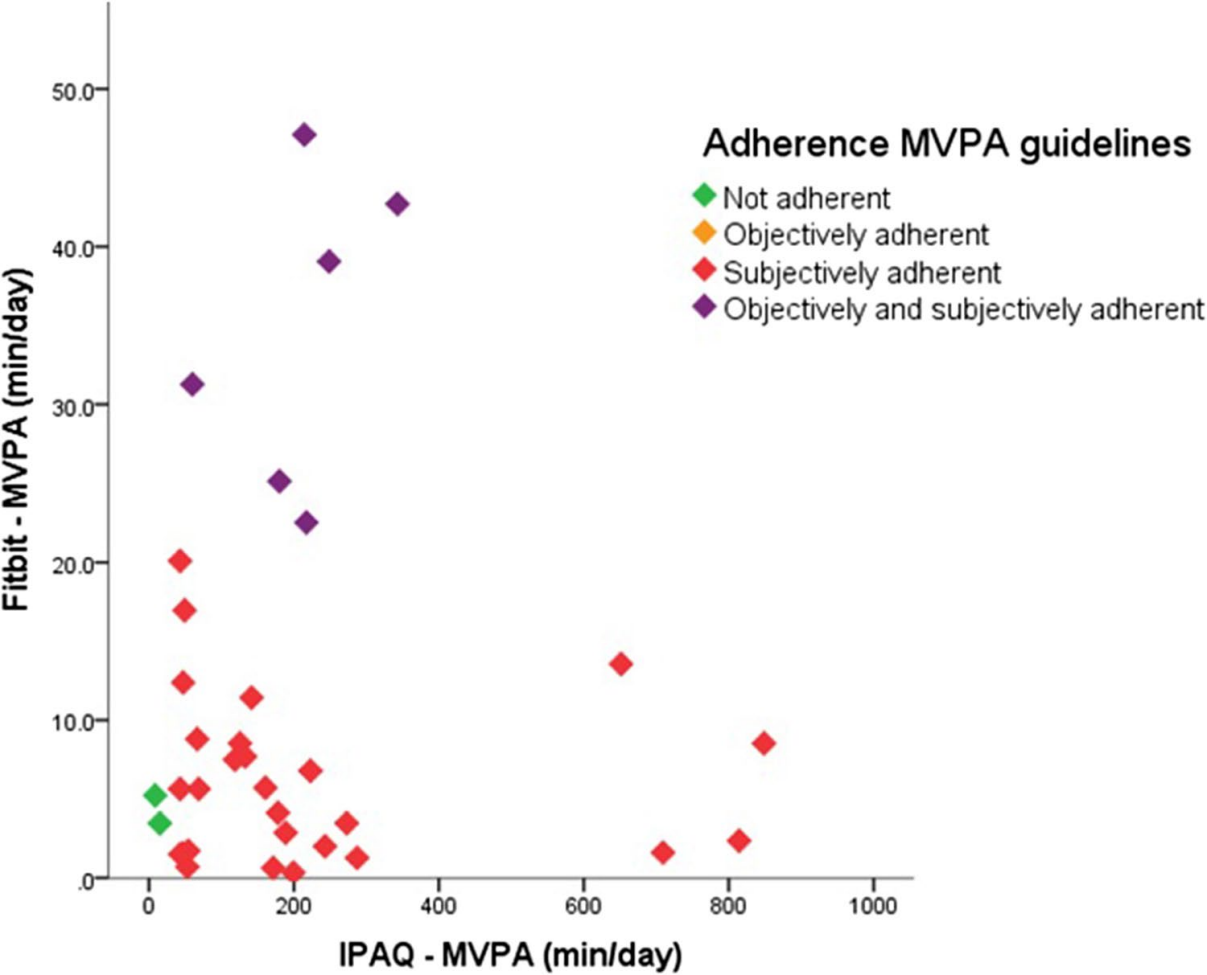
Self-reported and accelerometer-based MVPA in minutes per day

Twenty-five pre-surgery and ten post-surgery patients completed the IPAQ and had sufficient accelerometer data. Self-reported SB had a moderate positive correlation with accelerometer-based SB (Spearman’s rho: 0.455, *p* = 0.006). However, most participants underestimated their sedentary time, as accelerometer-measured sedentary time was higher than self-reported sedentary time (Fig. 3). Self-reported MVPA had a negligible non-significant positive correlation with accelerometer-based MVPA (Spearman’s rho: 0.072, *p* = 0.683). Overestimation of time spent in MVPA was seen in the entire study population (Fig. 4).

### Adherence PA Guidelines

Figure 4 shows the self-reported and accelerometer-measured minutes of MVPA per day per participant and the level of adherence, either subjectively and objectively, to MVPA guidelines. When self-reporting, the majority of participants (94.3%) is adherent to the PA guidelines of at least 150 min of MVPA per week, whereas sensor-based measurements point out that 17.1% of the study population complied with the PA guidelines (Table 4). Only six participants (17.1%) were both objectively and subjectively adherent to the MVPA guidelines. There are significant more post-surgery participants objectively adherent to the MVPA guidelines compared to pre-surgery participants (40% vs. 8%, *p* = 0.043).

**Table 4:** Self-reported and accelerometer-based adherence MVPA guidelines

	Total (*n* = 35)	Pre-surgery (*n* = 25)	Post-surgery (*n* = 10)	*p*
IPAQ—MVPA				
< 150 min/week, *N* (%)	2 (5.7)	2 (8.0)	0 (0.0)	
> 150 min/week, *N* (%)	33 (94.3)	23 (92.0)	10 (100.0)	1.000
Fitbit—MVPA				
< 150 min/week, *N* (%)	29 (82.9)	23 (92.0)	6 (60.0)	
> 150 min/week, *N* (%)	6 (17.1)	2 (8.0)	4 (40.0)	0.043

*MVPA* moderate-to-vigorous physical activity, *IPAQ* international physical activity questionnaire

## Discussion

### Main Findings

This study confirmed that (1) in general bariatric patients were not sufficiently active, rarely engage in MVPA bouts and are highly sedentary, with a significantly higher daily step count and significantly more minutes spent in MVPA and MVPA bouts in the post-surgery group compared to the pre-surgery group; (2) correlations between self-reported and accelerometer measurements of time spent in MVPA and SB were negligible and moderately significant, respectively; and (3) only 17.1% of the participants in this study met the recommend 150 MVPA minutes per week when objectively measured, while 94.3% met the guidelines according to self-reports.

Our results showed an enormous discrepancy between self-reported and sensor-based MVPA. All participants in our study overestimated their time spent in MVPA. This finding supports previous studies showing that bariatric patients overestimate their actual MVPA level [9, 14, 18]. In our study, pre-surgery participants overestimated their MVPA time with 197.6 min/day, while post-surgery participants overestimated their MVPA time with 194.8 min/day. Also Bond et al., Berglind et al. and Possmark et al. found such an overestimation, but contrary to our study, they found a greater overestimation post-surgery compared to pre-surgery [10, 14, 26].

We found a negligible correlation (Spearman’s rho: 0.072) between accelerometer-based and self-reported PA. This is consistent with the weak correlation (Spearman’s rho: 0.24) found in persons with a BMI > 30 kg/m^2^ by Warner et al. [9] and in line with previous work finding lower accuracy of objective and subjective PA measurements among persons with obesity [27].

Concerning the MVPA guidelines of at least 150 min per week, only 17.1% of the participants in our study was adherent in case of sensor-based measurements, compared to 94.3% by self-reports, which is consistent with the results of Bergh et al. (2017) (*n* = 112) [28]. They reported an adherence rate to the MVPA guidelines of 17.9% in case of sensor-based measurements versus 80.2% in case of self-reports.

The lack of agreement between self-reports and sensor-based PA measurements advocates not to rely on questionnaires as a reliable measure of PA. However, self-reports are of interest as they provide information concerning the level of awareness of patients regarding their physical (in)activity, which is the first important step of behavior change [29].

Most PA studies in bariatric patients assessed MVPA only [10, 14], which does not adequately illustrate other domains of physical behavior such as PAL and SB, which are considered independent relevant aspects for physical fitness and health [30, 31]. We extended earlier work as we measured those different dimensions of physical behavior simultaneously and in conjunction with self-reported measures.

Both the large between-subject variability and the variability of the three physical behavior domains (i.e. PAL, SB and MVPA) between and within subjects, as well as the discrepancy between self-reported and sensor-based measurements, provide support for objective physical behavior measurements and, through this, more personalized recommendations in bariatric care.

In our study, median MVPA time was 5.6 min per day (IQR: 1.7 – 10.6), while mean daily step count was 7403 ± 3243. This is at least remarkable, as it means that only a small percentage of daily step count was classified as MVPA (≥ 95 steps/min), and thus, the majority of daily step count is performed in a cadence < 95 steps/min. An explanation could be that most participants walked in a lower intensity cadence in general, which can be confirmed by the daily active minutes (median 154, IQR: 125 – 230). Moreover, it could be that the MVPA threshold is not feasible for people with morbid obesity. However, O’Brien et al. showed various models to accurately predict step rate thresholds for MVPA and revealed that BMI was excluded as predictor variable [32].

In our study, accumulated steps per day averaged 6736 ± 2947 in the pre-surgery group and 8778 ± 3470 in the group post-surgery. King et al. showed a similar significant increase in daily step count from pre- to post-surgery, from 7688 steps/day at baseline to 8959 steps/day 1 year post-surgery. However, it needs to be highlighted that King et al. conducted a longitudinal study, while our study had a cross-sectional design [33].

Our results show no significant changes in SB between the pre- and post-surgery group, which might be an indication that reduction of sedentary time and interrupting sedentary bouts requires attention in postoperative care, but this can only be said very carefully given our cross-sectional and, in this respect, restrictive study design. Our study population spent most waking hours sedentary (77%) and in prolonged periods (49%). Previous research by Chapman et al., Babineau et al. and Crisp et al. has demonstrated similar daily sedentary time, respectively 72, 75% and 77% [16, 20, 34]. There are no specific recommendations concerning sedentary behavior. However, the detrimental health effects of sitting in general and sitting in uninterrupted periods are increasingly recognized as clinically relevant and are independent of whether patients meet PA guidelines [30, 31]. Matthews et al. found a lower mortality in low-active US adults (adults who spent 68% of their time sedentary) of 18% and 42%, if 1 h of sedentary time was replaced with either light intensity PA or MVPA, respectively [35]. Since our population could be defined as ‘low-active’, this may provide an important advice for bariatric patients, that is: replace sedentary time with PA.

Participation in MVPA and MVPA bouts is an important contributing factor for successful weight loss, improvement of cardiovascular fitness and mental wellbeing after bariatric surgery [1–4]. On a group level, patients in this study participated 5.6 (IQR: 1.7 – 10.6) min per day in MVPA (0.5% of waking hours) and had only 0.8 (IQR: 0 – 5.3) MVPA bout minutes per day, indicating the need to increase MVPA levels in bariatric patients. Post-surgery patients spent significant more minutes per day in MVPA and MVPA bouts, possibly because patients were better able to engage in MVPA due to functional improvements or because patients understand its importance. Again, due to our cross-sectional study design, it is not possible to determine causality. Afshar et al. and Zabatiero et al. performed a longitudinal study in which they objectively measured pre- to post-surgery MVPA time and demonstrated unchanged MVPA levels in the first 6 months post-surgery [13, 36]. Afshar et al. reported a mean of 11.5 MVPA minutes per day in the pre-surgery group and 11.6 MVPA minutes per day in the post-surgery group [13], representing higher MVPA levels compared to our population, but still far lower than (about half of) the recommended ≥ 150 min per week [5], which indicates that the bariatric population remains physically inactive with no improvement in MVPA levels from pre- to post-surgery.

### Strengths and Limitations

A strength of our study is the 2 weeks of sensor-based measurements, which gives a more reliable representation of the PA measures compared to previous studies with a shorter measurement period [12–15, 33, 36], as behavioral changes due to accelerometer wear are negligible after a measurement period of 7 days [22]. An important limitation of our study is its cross-sectional study design and relatively small sample size. Due to our cross-sectional study design, we were not able to assess changes in physical behavior over time. Nevertheless, to answer our research questions, the cross-sectional study design was applicable as it was not the aim to study changes as result of the intervention in between. Another limitation is that Fitbit devices tend to overestimate steps in free-living settings [37]. This might have resulted in increased PA levels in our study sample than in real life, emphasizing even more the importance of objective monitoring. As walking limitations are common in the bariatric population and the Fitbit accelerometer does not register other non-step-based activities such as cycling and swimming, PA results might have been affected. The use of wearables with more advanced features (i.e. Garmin) could probably overcome this limitation. Moreover, as most pre-surgery participants were included in winter and most post-surgery participants were included in spring, PA levels between both groups may be further apart due to seasonal variations than we showed in our study. In future research, a sample distribution along the seasons should be encouraged. Despite the limitations, our results emphasize the need for sensor-based PA monitoring in routine clinical care as an objective measurement tool to quantify physical behavior and are used as input to come to personalized recommendations.

## Conclusion

The results of our study show that bariatric patients are not able to adequately assess MVPA and moderately their SB by self-reports, which stresses the importance to incorporate sensor-based PA measurements in routine bariatric care. We objectively investigated different physical activity domains (PAL, MVPA and SB) simultaneously and found that despite a relatively high daily step count, bariatric patients rarely engage in MVPA and are highly sedentary, especially in bouts.

## Supplementary Information

Below is the link to the electronic supplementary material.

Supplementary file1 (DOCX 25 kb)
