# Supplementary material for: The Need for Objective Physical Activity Measurements in Routine Bariatric Care
Source: Obes Surg. 2022 Jun 23;32(9):2975–86. doi: 10.1007/s11695-022-06165-y (PMC9392711; doi:10.1007/s11695-022-06165-y)
Supplement: Supplementary file 1 — Supplementary file1 (DOCX 25 kb) [file 11695_2022_6165_MOESM1_ESM.docx]

**SUPPLEMENTARY MATERIAL**

Appendix A: PA measures

**APPENDIX A. PA MEASURES**

**PAL** was expressed by 1) the daily step count calculated as the average number of steps per day of all the valid measurement days and 2) the active minutes per day, calculated from the mean number of minutes with a step count > 10 per minute and a heart rate > 0 per minute per day of all the valid measurement days.

**MVPA.** Minutes with a step count ≥ 95 were identified as MVPA [38 – 40]. MVPA was expressed as median total amount in min/day, median bout length in minutes and median number of bouts/day.

**MVPA bout.** Ten or more consecutive MVPA minutes (being step count ≥ 95) were considered as MVPA bout. If there is one minute with a step count ≥ 80 and < 95 in the bout, the bout will be continued. This minute is counted as a minute of the bout to prevent underestimation of MVPA bouts due to small changes in walking pace. A step count < 80 per minute is not counted as a minute of the bout. However, if this measurement is surrounded with a step count ≥ 95 per minute, there is no interruption of the bout (imagine the participant is waiting for a traffic light). It is not possible to have a bout < 10 minutes, due to the conditions in the Matlab algorithm. Moreover, a step count ≥ 95 per minute in at least 70% of the values is necessary to comply the definition of a MVPA bout.

**SB** was defined as a step count of 0 per minute and a heart rate > 0 per minute. SB was expressed as median total amount in min/day, mean bout length in minutes and mean number of bouts/day.

**SB bouts** are accumulated in 30 minutes and longer. Other criteria for a SB bout are a step count equal to 0 in ≥ 70% of the bout minutes and a maximum of 3 consecutive minutes with a step count > 0 and ≤ 10 per minute.
